# Archaeal Lineages within the Human Microbiome: Absent, Rare or Elusive?

**DOI:** 10.3390/life5021333

**Published:** 2015-05-05

**Authors:** Hans-Peter Horz

**Affiliations:** Division of Virology, Institute of Medical Microbiology, RWTH Aachen University Hospital, Pauwelsstrasse 30, 52057 Aachen, Germany; E-Mail: jhorz@ukaachen.de; Tel.: +49-241-8088573; Fax: +49-241-8082483

**Keywords:** human-associated archaea, human microbiome, periodontal disease, intestinal disorders, rare biosphere, anthropology of microbes

## Abstract

Archaea are well-recognized components of the human microbiome. However, they appear to be drastically underrepresented compared to the high diversity of bacterial taxa which can be found on various human anatomic sites, such as the gastrointestinal environment, the oral cavity and the skin. As our “microbial” view of the human body, including the methodological concepts used to describe them, has been traditionally biased towards bacteria, the question arises whether our current knowledge reflects the actual ratio of archaea *versus* bacteria or whether we have failed so far to unravel the full diversity of human-associated archaea. This review article hypothesizes that distinct archaeal lineages within humans exist, which still await our detection. First, previously unrecognized taxa might be quite common but they have eluded conventional detection methods. Two recent prime examples are described that demonstrate that this might be the case for specific archaeal lineages. Second, some archaeal taxa might be overlooked because they are rare and/or in low abundance. Evidence for this exists for a broad range of phylogenetic lineages, however we currently do not know whether these sporadically appearing organisms are mere transients or important members of the so called “rare biosphere” with probably basic ecosystem functions. Lastly, evidence exists that different human populations harbor different archaeal taxa and/or the abundance and activity of shared archaeal taxa may differ and thus their impact on the overall microbiome. This research line is rather unexplored and warrants further investigation. While not recapitulating exhaustively all studies on archaeal diversity in humans, this review highlights pertinent recent findings that show that the choice of appropriate methodological approaches and the consideration of different human populations may lead to the detection of archaeal lineages previously not associated with humans. This in turn will help understand variations found in the overall microbiomes from different individuals and ultimately may lead to the emergence of novel concepts/mechanisms impacting human health.

## 1. Archaea and the Human Microbiome 

The human body is a microbial driven supraorganism living in cross symbiosis with a myriad of different microorganisms which collectively are referred to as the human microbiome [1]. The term “microbiome” goes beyond a mere list of microbial taxa present in a given habit as it includes also the entire repertoire of genes these microbes are equipped with. This is a non-trivial definition considering the plasticity of microbial genomes. Microorganisms can undergo extensive horizontal gene transfer forming a multitude of diversifying strains which share a common core genome but otherwise differ strongly by the types and amount of their accessory genes [2]. With the advent of next-generation sequencing technologies a number of microbiome studies have accumulated in literature, however they are performed with somewhat different emphasis. First, many studies dealing with the microbiome are based on an amplicon phylotyping approach in which 16S RNA gene inventories are generated [3,4]. While this approach allows the rapid determination of species occurrence and abundance in an efficient way for numerous samples simultaneously, it neglects, that identical species found in two different microbiomes may differ substantially in their functional capacities. Second, most studies focus only on bacteria while ignoring the domain *Archaea*, notwithstanding the fact that the microbiome also includes eukaryotic microorganisms and viruses as consistent components [4,5,6]. Intra- but also inter-domain interactions are usual, all of which may affect our health in numerous ways, negatively and positively. Therefore, valid conclusions regarding human health based on the bacterial microbiome alone can be misleading. 

Since archaea have long been ignored in medical microbiology, it is quite likely that we have still not encompassed the full breadth of archaeal diversity within humans. Clearly, bacteria constitute the bulk of the human microbiome and the most common and abundant archaeal species therein are probably also well known to us (as discussed in the next paragraphs). Therefore, one can assume that additional, not yet recognized archaeal taxa, if existing, are probably rather uncommon. This raises the question, whether it is necessary to identify all taxa, even if they are rare and/or only in very low abundance. Would it not suffice to deal only with the persistent and reasonable abundant species known so far for understanding the impact of the human microbiome on health and disease? This question cannot be answered yet, because with our current knowledge, we cannot even say with certainty whether some archaeal taxa are truly absent, rare or relatively common but just elusive, using traditional detection methods. This implies that because of the absence of appropriate tools, distinct archaeal organisms with unique metabolic capacities are still awaiting our discovery. In fact, two major archaeal lineages, which are described in a later chapter, with reasonable abundance in the human microbiome have been described only recently with modified PCR-based approaches. Apart from this, it is also worthwhile to look for rare members, if we adhere to the concept of the “rare biosphere” [7]. Long-term series analysis have shown that species or taxa typically found at very low abundance can become prevalent from time to time and it is believed that their activity then might provide an essential function to the community [8]. Hence, it is possible from an ecological perspective that by moving in and out of the “rare biosphere such “blooming” taxa are important for community resilience and stability, including responses to and recovery from disturbances [8]. Conceivably, many rare taxa may not have been found yet because we have not sampled the right individual at the right “blooming” time of a rare taxon. Of course the concept of the “rare biosphere” applies to both domains, *Bacteria* and *Archaea*. However, owing to their unique properties archaea can be considered key species in an environment otherwise dominated by bacteria, such as the human microbiome [9]. This means, while there is a more or less high functional diversity across many bacterial taxa, which can replace each other and thereby maintain ecosystem functions, this is much less likely for the few human archaeal taxa. Changes in the type of archaeal species or in their metabolic activities even if remaining at low abundance may have profound effects on the entire microbiome and thus could lead at least indirectly to the predisposition for various diseases.

## 2. The Medical Importance of the “Common” Archaea

Traditionally, our perception about human archaea and particularly archaeal diversity has been lagging behind compared to the knowledge that we have about human-associated bacteria. This is evident from the fact that the list of newly described archaeal lineages associated with the human body has only slowly increased in recent years. For many decades knowledge of human archaea was restricted to three cultivable organisms from the phylum *Euryarchaeota*: *Methanobrevibacter smithii* and *Methanosphaera stadtmanae*, which are primarily found in the human gut system [10,11] and *Methanobrevibacter oralis*, which thrives in various niches within the oral cavity [12]. As methane-producers (methanogens), these organisms were already recognized for their unique metabolic capacities, however their discovery in humans predate the time when Carl Woese’s “three-primary domain-concept” [13] was scientifically accepted, and consequently methanogenic organisms were then still addressed as “bacteria” [14,15]. The prevailing official taxonomic names, such as “*Methanobacteriales*” or “*Methanobrevibacter*” represent taxonomical relicts of this time. Evidence for the existence of methanogenic organisms in the human gut was also provided through breath methane measurements. Furthermore, a number of studies indicated associations between gut methanogens and intestinal disorders [16,17,18,19,20], although till today a distinction between cause and effect cannot be made. Based on these and later investigations relying on elaborate molecular based detection and quantification methods, the above mentioned archaeal species were frequently identified in a multitude of gut and oral samples and a possible link to various diseases (in particular periodontitis and dental root canal infections) could be established and has been discussed ever since [21,22,23,24,25]. Methanogens as the causal agents of disease have never been proven so far and one reason for this is due to the fact that the cultivation of methanogens and the study of their physiology is tedious and cumbersome. Even if successfully grown, this is usually done under artificial laboratory conditions (*i.e*., 80% H_2_ and 20% CO_2_ atmosphere). Therefore conclusions regarding true *in vivo* activity and interactions with other microorganisms at oral sites of infection or in the gut are difficult to draw if not entirely impossible.

Nonetheless, the fact remains that methanogens have a unique metabolism. Apart from the formation of methane as an end-product, they use a number of distinct substrates as energy and carbon source, such as methanol, methylamines, methyl-sulfides, acetate, CO_2_, and notably also molecular hydrogen (H_2_) provided by hydrolytic and fermentative bacteria. While otherwise blocking ongoing fermentation, the removal of H_2_ positions the methanogenic archaea at the bottom of the anaerobic food chain which in turn allows an energetically effective and comprehensive degradation of organic matter in an anaerobic environment [26]. This syntrophic relationship is generally referred to as “interspecies hydrogen transfer” and it provides ideal conditions to flourish for the involved microbial communities [27]. Consequently, it is plausible to assume that methanogenic activity in periodontal pockets (mostly *M. oralis*) promotes progression of periodontal disease even if they are not the real causative agent(s) of disease or do not express classical virulence factors. Likewise, based on the same metabolic activity methanogens in the human gut (*i.e*., *M. smithii*) can be considered key species *par excellence*. Even in the absence of an infection, syntrophic partners involved in interspecies hydrogen transfer, their preferred substrates and resulting end-products may have an impact directly or indirectly on the host physiology and ultimately on health status, as for example colon cancer, inflammatory bowel diseases, cardiovascular disease and obesity [28,29]. This important role is also borne out by the fact that the genus *Methanobrevibacter* has been described as a component of one of the three so-called “enterotype networks” [30]. Even if likely more complex than initially thought [31], co-occurrence in this network likely indicates that interspecies hydrogen transfer is an important interaction in the human gut. The fact that the second “enterotype network”, lacking *Methanobrevibacter*, contains *Desulfovibrio*, one of the few bacterial genera, also capable of H_2_-consumption underscores the potential importance of interspecies hydrogen transfer in the human gut [30]. 

## 3. Hitherto Elusive Archaea and Potential for Human Health

Archaea have traditionally been divided into five phyla, namely *Crenarchaeota*, *Euryarchaeota*, *Korarchaeota*, *Nanoarchaeota*, and *Thaumarchaeota*. Based on the increasing wealth of whole genome data (mainly from environmental isolates), the archaeal phylogeny has been revisited recently: the four groups *Korarchaeota*, *Crenarchaeota*, *Thaumarchaeota* and the newly proposed *Aigarchaeota* have been comprised into one superphylum (the so-called TACK-superphylum) to the exclusion of *Euryarchaeota* and *Nanoarchaeota* [32]. In addition, these novel genomic insights have also challenged the three-primary domain-concept as increasing evidence exists, that the eukaryotic lineage emerged from genomic fusion between an archaeal lineage of the TACK-Superphylum with bacterial lineages [32]. Although still different evolutionary scenarios are under discussion at the moment [33], each one conflicting somewhat with the others, new exciting insights can be expected in the foreseeable future with ongoing functional and genomic studies. 

Except for *Korarchaeota* and *Nanoarchaeota*, members of the other phyla have been identified in the human body, either as cultured isolates, or cultured in a consortium of microbes, or identified solely by 16S rRNA genes, or protein coding genes, such as *mcrA* and *amoA* gene sequences. For a detailed overview the reader is referred to the article by Gaci *et al*. (2014) [34]. 

Only after a gap of approximately 30 years, when the first methanogens in humans were described, evidence for a novel group of methanogens within the phylum *Euryarchaeota* but distinct from the “*Methanobacteriales*” (the order which contains the “classical” human methanogens), was found in the human gut [35,36]. Further discovery was based on archaeal 16S rRNA gene analysis, which indicated a common line of descent with members of the *Thermoplasmatales* [36,37]. Intriguingly, unique sequences of the *mcrA* gene encoding for an enzymatic key step in methanogenesis were found in the very same samples, but not in samples that tested negative for the novel 16S rRNA gene sequences [37]. This congruency gave rise to the speculation of the existence of a yet uncharacterized group of methanogens (along with the then recognized six orders of methanogens, namely *Methanobacteriales*, *Methanococcales*, *Methanomicrobiales*, *Methanosarcinales*, *Methanopyrales*, and *Methanocellales*). The prevalence of this novel archaeal group was found to range from 10% to 40%, depending on age [37]. 

Having worked on oral microbiology research projects at that time, our group immediately reasoned that such novel organisms could be present in the oral cavity as well, which was also supported by a single reported clone sequence [38]. In fact, based on PCR primer systems with intended target specificity for this novel group, we were soon able to identify corresponding 16S rRNA gene and *mcrA* gene in 10% of tested periodontal samples [39]. Interestingly, the oral sequences were related to, but distinct from the gut-derived sequences. This is compatible with an earlier observation, that both human compartments harbor predominantly different types of archaea, a fact that also applies largely to bacteria [40,41]. Hence, our increased awareness of additional archaeal lineages as being part of the human microbiome was only possible with the application of refined primer systems. 

Soon thereafter the successful isolation of the first corresponding organism from the human gut, named *Methanomassiliicoccus luminyensis* was reported, and the methanogenic phenotype confirmed [42]. Further genome wide studies gave fascinating insights into the unique biology of this novel archaeal group for which now the official name *Methanomassiliicoccales* has been validated. It belongs to the seventh order of methanogenic archaea, of the class *Thermoplasma* and neighbors the order *Thermoplasmatales* [43,44,45,46]. What are the metabolic properties of these novel archaea, and how can they affect human health? First, members of this novel lineage are dependent on H_2_ and can, like other gut-associated methanogens, use methanol for methanogenesis. However, genomic studies revealed some peculiarities, and besides the complete absence of the CO_2_-reduction/methyl-oxidation pathways these organisms have the capacity to encode an unusual/rare proteinogenic amino acid, pyrrolysine (Pyl) [47]. This unique feature which is shared only with a few bacteria and some members of the family *Methanosarcinaceae* is not only exciting from an evolutionary perspective, but likely has an important impact on human health. The reason for this is that because methylotrophic methanogenesis based on methylated amines, such as monomethylamine (MMA), dimethylamine (DMA) or trimethylamine (TMA) only works in the presence of pyrrolysine in the active catalytic site [34]. In fact growth experiments based on those methylated amines demonstrated, that *M. luminyensis* was able to effectively use these methylated amines as electron acceptors under the formation of methane [48]. Maybe better known as the substance responsible for the characteristic odor of rotting fish, TMA is also a natural by-product of diet metabolism in the gut, and a link between its oxidized form and cardio-vascular diseases has been recently established [49,50,51]. It is plausible to assume that if members of this novel archaeal organisms are present in the gut they might contribute to reduce the TMA levels and thus possibly reduce the risk for such diseases. Although at this point still somewhat visionary an innovative concept to use this species as a form of “archaebiotics” for individuals not harboring this species has recently been proposed [48]. This example demonstrates the potent physiological importance of a hitherto unknown archaeal lineage in the human gut.

Another example refers to a distinct archaeal lineage recently found on the skin of humans with comparably high abundance for the first time. Here, with a prevalence of 100% (13 tested individuals) the archaeal 16S rRNA gene copies comprised 0.60% on average and up to 4.2% of the prokaryotic skin microbiome [52]. Most of the gene signatures analyzed belonged to *Thaumarchaeota*, a recently proposed phylum including designated ammonia-oxidizers, which have been re-classified from a distinct lineage of mesophilic *Crenarchaeota* [53]. In fact, besides the 16S rRNA gene sequences *amoA* gene sequences (encoding for the key enzyme ammonium monooxygenase) also affiliated to *Thaumarchaeota* were identified within the same samples affirming the existence of ammonia-oxidizing archaea as natural component of the human skin [52]. This finding is intriguing, since until recently archaea were not believed to be colonizers of the human skin (our largest organ) in reasonable amounts [54,55]. Furthermore, as chemolithotrophic ammonia oxidizers these organisms could influence the pH regulation which is the natural protective layer of the human skin [52]. Overall the perception of archaea as a component of the skin ecosystem could advance our understanding of the balance between host and skin microbiome and help get novel insights into the microbial involvement in human skin disorders.

Interestingly, identical sequences were also found in intensive care units from two hospitals and it has been speculated that this source of contamination came from the humans themselves carrying respective organisms on their skin [52]. This raises the question if members of this novel archaeal lineage could be of clinical importance once translocated to other human body habitats. At least their existence has also been confirmed in the human gut, though with low abundance [56]. 

At the time when archaeal ammonia oxidation was first discovered in natural environments [57] and conserved *amoA* gene primers for this archaeal group were published [58] our group was prompted to test the possible presence of this unique archaeal lineage in the oral cavity. No such sequences were found at that time, but failure to do so, could be due to primer mismatches to the according template regions. In fact, while the reverse primers showed no mismatches we observed that the forward primer showed several mismatches to those archaeal sequence types, recently found in the skin microbiome [52,58]. The existence of these archaeal lineages on skin and in low abundance in the human gut makes the oral cavity a likely additional habitat and we speculate that evidence for this will be provided with appropriate PCR primer systems in the near future. 

## 4. Are There yet More Archaea Awaiting Detection in Humans?

The two examples outlined above demonstrate, that distinct archaeal lineages with reasonable abundance and potential medical importance have escaped our awareness for very long time. Therefore the title question of this chapter is certainly warranted and automatically leads to another question: what keeps us from recognizing archaeal organisms in the human microbiome? One reason for this problem is, that traditionally our “microbial” view of the human body is largely focused on bacteria and not on archaea, as are the methodological concepts, which are highly biased towards bacteria. Cultivation methods from clinical samples in routine diagnosis are specifically adapted to bacteria, making it impossible to even accidently find archaeal organisms in liquid or on solid media, even if present in reasonable amounts in the initial inoculum. Furthermore, cultivation-independent approaches largely fail, if conditions stay adjusted to bacteria. DNA-extraction protocols developed for lysis of bacterial cell walls for example fail, if ignoring the unique rigid cell wall features of archaea. Many archaeal cell walls, including those of the “common” human archaea are composed of an electron-dense pseudomurein-layer as well as an additional second layer of heteropolysaccharides [59,60]. This means that DNA isolation using protocols optimized for bacterial cell lysis may be inefficient for archaeal cells. 

In fact, when using a modified DNA extraction protocol on stool samples from 700 individuals, the prominent gut archaeon *M. smithii* was identified with a significantly higher prevalence of 95% than previously estimated [61]. Hence, it is quite likely to identify previously undetected archaeal taxa or to find known taxa in higher abundance, when using appropriate or modified extraction protocols. The use of appropriate PCR-primer systems is a logical next step for improved detection of novel taxa, and this obvious requirement has already been illustrated with the two examples given in the previous chapter. 

Irrespective of this, when it comes to identify novel archaeal lineages as true components of the rare biosphere, we need more than a single snapshot and samples of an individual have to be analyzed in time serial datasets. These organisms might not be detected even with improved DNA extraction and PCR protocols, since their abundance is simply below the detection method. Recent analysis of the temporal variation of the human microbiome using next-generation technologies have in fact indicated that there are many taxa (even quite common bacterial taxa) that are persistent but non-permanent community members [55]. This means that taxa may vanish from time to time below the detection limit but never get entirely “lost” from the human ecosystem. Fluctuations in abundance may apply to all microbiome members, *i.e*., to those present in comparably high proportions, but also to those present in rather low proportions. This in turn demands for more efforts to verify whether rather infrequently described archaeal taxa based on 16S rRNA gene analysis represent possible candidates of the rare archaeal biosphere. This includes a phylogenetic broad range of methanogenic and non-methanogenic species mainly from human gut, such as *Methanoculleus chikugoensis*, *Methanosarcina mazei*, *Halorubrum* spp., *Haloplanus* sp., *Halococcus* sp. *Natronorubrum* sp., *Sulfolobus* spp., and *Nitrososphaera* spp. (reviewed in detail by Gaci *et al*. [34]). Either these organisms are just transient members, e.g., through ingestion of food, and might not even be viable, or they are true colonizers, yet rare and subtle (but still important) members of the microbiome. Identification of such archaeal species and describing their dynamics may help understand the underlying patterns that may lead to their “blooming” in and out of the rare biosphere. This includes resulting variations in the overall microbiome caused by such fluctuations and possible associations with pathological states. 

Infrequent detection of archaeal taxa may have another reason yet to be explored, and this relates to the selection of the individuals that have been sampled. In most studies, the microbiome of Caucasian individuals has been investigated, and only recently have studies considered that individuals from different human populations may differ in their microbiomes [62,63] Hence, with the emerging view of the “anthropology of microbes” [64], it is of particular interest, to know whether the microbiome is influenced more by intrinsic/internal factors (including phylogeny, vertical transmission, host physiology, *etc*.) or more by extrinsic/external factors (such as diet, environment, geography, *etc*.); While this research line is still in its infancy, a number of studies have already indicated that the microbiome from different human populations of different ethnic and geographic origin differ either with respect to species composition or proportions of different taxa [4,65,66,67]. As many analyses have primarily focused on bacteria, again, knowledge regarding archaea lags behind. There are, however, some indications in literature showing that archaeal taxa or their abundance and activity differ between human populations making the “anthropology of archaea” an interesting field of study. For instance, unique archaeal sequences related to halobacteria have been detected previously in the gut of healthy Koreans [68]. Co-identification of the same group of organisms in salt-fermented seafood suggests the specific diet of these individuals as the reason for the existence of this unique archaeal group in the gut [68]. Furthermore, the prominent gut archaeon *M. smithii* has been reported to differ significantly among different individuals from Russia, China, Denmark and USA [69]. In accordance with this observation, earlier breath methane measurements also indicated significant differences in methane production in the gut of staff members of two Canadian hospitals from different geographic origins (*i.e*., Caucasians, Africans, Orientals and Indians). This could be due to different proportions and/or activities, but also due to different types of methanogens in the gut of the tested individuals [70]. Similar observations stem from breath methane measurements from four different human populations in South Africa. Again, methane production in the human colon showed significant interethnic differences. Interestingly, these four populations are at different risks for bowel cancer and other colonic diseases, but no correlation between these risks and the methane production was observed [71]. Likewise, another study showed, that Hawaiians and Caucasians produce more colonic methane than three different Asian groups, but again these differences did not correlate with risk of colon cancer among these ethnic populations [72]. In a more recent study, also motivated by the existing differential risk for colorectal cancer among ethnic groups, genetic fingerprint analysis (*i.e*., terminal restriction fragment length analysis) revealed significantly different types of methanogens in native Africans, African Americans and European Americans [73]. Native Africans, who have a lower risk of sporadic colorectal cancer produce more colonic methane and are characterized by higher methanogenic diversity than the American and European Africans, which again is probably due to different diets [73]. The data show that there are no clear but rather complex relationships between the production of colonic gases, such as methane, H_2_ or hydrogen sulfide, and various human bowel disorders. Furthermore, there is the need for standardized breath gas measurements combined with the ever-improving next-generation sequencing technologies to establish the link between type and proportions of methanogens within the overall gut microbiome and methane production. Increasing knowledge in this area will provide novel strategies to prevent, diagnose or manage numerous colonic disorders [74]. Taken together, different human populations are characterized by different archaeal taxa, or by different amounts and/ or activity of shared archaeal taxa in the gut. This research field is rather unexplored and studies addressing this issue will bring novel insights not only from an anthropological but also medical perspective. 

## 5. Final remark

The study of human archaea has gained increasing interest. As indicated by most recent findings, the detection of archaeal taxa previously unrecognized within humans may lead to novel concepts or mechanisms impacting human health. The sooner the impact or practical applications of newly identified lineages will be better understood, the larger the continued scientific efforts will be to further reveal the true archaeal diversity within humans. Finally, controversies that exist between studies based on bacterial microbiome analysis alone may be reduced when complemented by data from the missing link—the *Archaea*.

## References

[1] Turnbaugh P.J., Ley R.E., Hamady M., Fraser-Liggett C.M., Knight R., Gordon J.I. (2007). The Human Microbiome Project. Nature.

[2] Medini D., Donati C., Tettelin H., Masignani V., Rappuoli R. (2005). The microbial pan-genome. Curr. Opin. Genet. Dev..

[3] Dewhirst F.E., Chen T., Izard J., Paster B.J., Tanner A.C.R., Yu W.-H., Lakshmanan A., Wade W.G. (2010). The Human Oral Microbiome. J. Bacteriol..

[4] Rehman A., Rausch P., Wang J., Skieceviciene J., Kiudelis G., Bhagalia K., Amarapurkar D., Kupcinskas L., Schreiber S., Rosenstiel P. (2015). Geographical patterns of the standing and active human gut microbiome in health and IBD. Gut.

[5] Parfrey L.W., Walters W.A., Knight R. (2011). Microbial Eukaryotes in the Human Microbiome: Ecology, Evolution, and Future Directions. Front. Microbiol..

[6] Pride D.T., Salzman J., Haynes M., Rohwer F., Davis-Long C., White R.A., Loomer P., Armitage G.C., Relman D.A. (2012). Evidence of a robust resident bacteriophage population revealed through analysis of the human salivary virome. ISME J..

[7] Sogin M.L., Morrison H.G., Huber J.A., Welch D.M., Huse S.M., Neal P.R., Arrieta J.M., Herndl G.J. (2006). Microbial diversity in the deep sea and the underexplored “rare biosphere”. Proc. Natl. Acad. Sci. USA.

[8] Shade A., Gilbert J.A. (2015). Temporal patterns of rarity provide a more complete view of microbial diversity. Trends Microbiol..

[9] Horz H.-P., Conrads G. (2011). Methanogenic *Archaea* and oral infections—ways to unravel the black box. J. Oral Microbiol..

[10] Miller T.L., Wolin M.J., de Macario E.C., Macario A.J. (1982). Isolation of *Methanobrevibacter smithii* from human feces. Appl. Environ. Microbiol..

[11] Fricke W.F., Seedorf H., Henne A., Krüer M., Liesegang H., Hedderich R., Gottschalk G., Thauer R.K. (2006). The genome sequence of *Methanosphaera stadtmanae* reveals why this human intestinal archaeon is restricted to methanol and H_2_ for methane formation and ATP synthesis. J. Bacteriol..

[12] Ferrari A., Brusa T., Rutili A., Canzi E., Biavati B. (1994). Isolation and characterization of Methanobrevibacter oralis sp. nov.. Curr. Microbiol..

[13] Woese C.R., Kandler O., Wheelis M.L. (1990). Towards a natural system of organisms: Proposal for the domains Archaea, Bacteria, and Eucarya. Proc. Natl. Acad. Sci. USA.

[14] Belay N., Johnson R., Rajagopal B.S., de Macario E.C., Daniels L. (1988). Methanogenic bacteria from human dental plaque. Appl. Environ. Microbiol..

[15] Belay N., Mukhopadhyay B., de Macario E.C., Galask R., Daniels L. (1990). Methanogenic bacteria in human vaginal samples. J. Clin. Microbiol..

[16] Lee K.-M., Paik C.-N., Chung W.C., Yang J.-M., Choi M.-G. (2013). Breath methane positivity is more common and higher in patients with objectively proven delayed transit constipation. Eur. J. Gastroenterol. Hepatol..

[17] Weaver G.A., Krause J.A., Miller T.L., Wolin M.J. (1986). Incidence of methanogenic bacteria in a sigmoidoscopy population: An association of methanogenic bacteria and diverticulosis. Gut.

[18] Haines A., Dilawari J., Metz G., Blendis L., Wiggins H. (1977). Breath-methane in patients with cancer of the large bowel. The Lancet.

[19] Zhang H., DiBaise J.K., Zuccolo A., Kudrna D., Braidotti M., Yu Y., Parameswaran P., Crowell M.D., Wing R., Rittmann B.E., Krajmalnik-Brown R. (2009). Human gut microbiota in obesity and after gastric bypass. Proc. Natl. Acad. Sci. USA.

[20] Piqué J.M., Pallarés M., Cusó E., Vilar-Bonet J., Gassull M.A. (1984). Methane production and colon cancer. Gastroenterology.

[21] Conwaydemacario E., Macario A. (2009). Methanogenic archaea in health and disease: A novel paradigm of microbial pathogenesis. Int. J. Med. Microbiol..

[22] Lepp P.W., Brinig M.M., Ouverney C.C., Palm K., Armitage G.C., Relman D.A. (2004). Methanogenic Archaea and human periodontal disease. Proc. Natl. Acad. Sci. USA.

[23] Vianna M.E., Conrads G., Gomes B.P.F.A., Horz H.P. (2006). Identification and Quantification of Archaea Involved in Primary Endodontic Infections. J. Clin. Microbiol..

[24] Vianna M.E., Holtgraewe S., Seyfarth I., Conrads G., Horz H.P. (2008). Quantitative Analysis of Three Hydrogenotrophic Microbial Groups, Methanogenic Archaea, Sulfate-Reducing Bacteria, and Acetogenic Bacteria, within Plaque Biofilms Associated with Human Periodontal Disease. J. Bacteriol..

[25] Horz H.-P., Conrads G. (2010). The Discussion Goes on: What Is the Role of Euryarchaeota in Humans?. Archaea.

[26] Thauer R.K., Kaster A.-K., Seedorf H., Buckel W., Hedderich R. (2008). Methanogenic archaea: Ecologically relevant differences in energy conservation. Nat. Rev. Microbiol..

[27] Schink B. (1997). Energetics of syntrophic cooperation in methanogenic degradation. Microbiol. Mol. Biol. Rev..

[28] Eckburg P.B., Lepp P.W., Relman D.A. (2003). Archaea and Their Potential Role in Human Disease. Infect. Immun..

[29] Cavicchioli R., Curmi P.M.G., Saunders N., Thomas T. (2003). Pathogenic archaea: Do they exist?. BioEssays.

[30] Arumugam M., Raes J., Pelletier E., Le Paslier D., Yamada T., Mende D.R., Fernandes G.R., Tap J., Bruls T., Batto J.-M. (2011). Enterotypes of the human gut microbiome. Nature.

[31] Jeffery I.B., Claesson M.J., O’Toole P.W., Shanahan F. (2012). Categorization of the gut microbiota: enterotypes or gradients?. Nat. Rev. Microbiol..

[32] Guy L., Ettema T.J.G. (2011). The archaeal “TACK” superphylum and the origin of eukaryotes. Trends Microbiol..

[33] Petitjean C., Deschamps P., Lopez-Garcia P., Moreira D. (2015). Rooting the Domain Archaea by Phylogenomic Analysis Supports the Foundation of the New Kingdom Proteoarchaeota. Genome Biol. Evol..

[34] Gaci N., Borrel G., Tottey W., O’Toole P.W., Brugère J.-F. (2014). Archaea and the human gut: New beginning of an old story. World J. Gastroenterol..

[35] Scanlan P.D., Shanahan F., Marchesi J.R. (2008). Human methanogen diversity and incidence in healthy and diseased colonic groups using mcrA gene analysis. BMC Microbiol..

[36] Mihajlovski A., Alric M., Brugère J.-F. (2008). A putative new order of methanogenic Archaea inhabiting the human gut, as revealed by molecular analyses of the mcrA gene. Res. Microbiol..

[37] Mihajlovski A., Doré J., Levenez F., Alric M., Brugère J.-F. (2010). Molecular evaluation of the human gut methanogenic archaeal microbiota reveals an age-associated increase of the diversity. Environ. Microbiol. Rep..

[38] Li C.L., Liu D.L., Jiang Y.T., Zhou Y.B., Zhang M.Z., Jiang W., Liu B., Liang J.P. (2009). Prevalence and molecular diversity of Archaea in subgingival pockets of periodontitis patients. Oral Microbiol. Immunol..

[39] Horz H.-P., Seyfarth I., Conrads G. (2012). McrA and 16S rRNA gene analysis suggests a novel lineage of Archaea phylogenetically affiliated with *Thermoplasmatales* in human subgingival plaque. Anaerobe.

[40] Maukonen J., Mättö J., Suihko M.-L., Saarela M. (2008). Intra-individual diversity and similarity of salivary and faecal microbiota. J. Med. Microbiol..

[41] Rajilić-Stojanović M., Smidt H., de Vos W.M. (2007). Diversity of the human gastrointestinal tract microbiota revisited. Environ. Microbiol..

[42] Dridi B., Fardeau M.-L., Ollivier B., Raoult D., Drancourt M. (2012). *Methanomassiliicoccus luminyensis* gen. nov., sp. nov., a methanogenic archaeon isolated from human faeces. Int. J. Syst. Evol. Microbiol..

[43] Borrel G., Harris H.M.B., Parisot N., Gaci N., Tottey W., Mihajlovski A., Deane J., Gribaldo S., Bardot O., Peyretaillade E. (2013). Genome Sequence of “Candidatus *Methanomassiliicoccus intestinalis*” Issoire-Mx1, a Third Thermoplasmatales-Related Methanogenic Archaeon from Human Feces. Genome Announc..

[44] Borrel G., Harris H.M.B., Tottey W., Mihajlovski A., Parisot N., Peyretaillade E., Peyret P., Gribaldo S., O’Toole P.W., Brugère J.-F. (2012). Genome sequence of “Candidatus *Methanomethylophilus*
*alvus*” Mx1201, a methanogenic archaeon from the human gut belonging to a seventh order of methanogens. J. Bacteriol..

[45] Borrel G., Parisot N., Harris H.M.B., Peyretaillade E., Gaci N., Tottey W., Bardot O., Raymann K., Gribaldo S., Peyret P., O’Toole P.W., Brugère J.-F. (2014). Comparative genomics highlights the unique biology of *Methanomassiliicoccales*, a Thermoplasmatales-related seventh order of methanogenic archaea that encodes pyrrolysine. BMC Genomics.

[46] Borrel G., O’Toole P.W., Harris H.M.B., Peyret P., Brugère J.-F., Gribaldo S. (2013). Phylogenomic data support a seventh order of Methylotrophic methanogens and provide insights into the evolution of Methanogenesis. Genome Biol. Evol..

[47] Borrel G., Gaci N., Peyret P., O’Toole P.W., Gribaldo S., Brugère J.-F. (2014). Unique Characteristics of the Pyrrolysine System in the 7th Order of Methanogens: Implications for the Evolution of a Genetic Code Expansion Cassette. Archaea.

[48] Brugère J.-F., Borrel G., Gaci N., Tottey W., O’Toole P.W., Malpuech-Brugère C. (2014). Archaebiotics: Proposed therapeutic use of archaea to prevent trimethylaminuria and cardiovascular disease. Gut Microbes.

[49] Zhang A.Q., Mitchell S.C., Smith R.L. (1999). Dietary precursors of trimethylamine in man: A pilot study. Food Chem. Toxicol..

[50] Koeth R.A., Wang Z., Levison B.S., Buffa J.A., Org E., Sheehy B.T., Britt E.B., Fu X., Wu Y., Li L. (2013). Intestinal microbiota metabolism of L-carnitine, a nutrient in red meat, promotes atherosclerosis. Nat. Med..

[51] Wang Z., Tang W.H.W., Buffa J.A., Fu X., Britt E.B., Koeth R.A., Levison B.S., Fan Y., Wu Y., Hazen S.L. (2014). Prognostic value of choline and betaine depends on intestinal microbiota-generated metabolite trimethylamine-N-oxide. Eur. Heart J..

[52] Probst A.J., Auerbach A.K., Moissl-Eichinger C. (2013). Archaea on Human Skin. PLoS ONE.

[53] Brochier-Armanet C., Boussau B., Gribaldo S., Forterre P. (2008). Mesophilic Crenarchaeota: Proposal for a third archaeal phylum, the *Thaumarchaeota*. Nat. Rev. Microbiol..

[54] Grice E.A., Segre J.A. (2011). The skin microbiome. Nat. Rev. Microbiol..

[55] Caporaso J.G., Lauber C.L., Costello E.K., Berg-Lyons D., Gonzalez A., Stombaugh J., Knights D., Gajer P., Ravel J., Fierer N. (2011). Moving pictures of the human microbiome. Genome Biol..

[56] Hoffmann C., Dollive S., Grunberg S., Chen J., Li H., Wu G.D., Lewis J.D., Bushman F.D. (2013). Archaea and fungi of the human gut microbiome: Correlations with diet and bacterial residents. PLoS ONE.

[57] Könneke M., Bernhard A.E., de la Torre J.R., Walker C.B., Waterbury J.B., Stahl D.A. (2005). Isolation of an autotrophic ammonia-oxidizing marine archaeon. Nature.

[58] Francis C.A., Roberts K.J., Beman J.M., Santoro A.E., Oakley B.B. (2005). Ubiquity and diversity of ammonia-oxidizing archaea in water columns and sediments of the ocean. Proc. Natl. Acad. Sci. USA.

[59] Kandler O., König H. (1998). Cell wall polymers in Archaea (Archaebacteria). Cell. Mol. Life Sci..

[60] Kandler O., König H. (1978). Chemical composition of the peptidoglycan-free cell walls of methanogenic bacteria. Arch. Microbiol..

[61] Dridi B., Henry M., El Khéchine A., Raoult D., Drancourt M. (2009). High prevalence of *Methanobrevibacter smithii* and *Methanosphaera stadtmanae* detected in the human gut using an improved DNA detection protocol. PLoS ONE.

[62] Human Microbiome Project Consortium (2012). Structure, function and diversity of the healthy human microbiome. Nature.

[63] Yatsunenko T., Rey F.E., Manary M.J., Trehan I., Dominguez-Bello M.G., Contreras M., Magris M., Hidalgo G., Baldassano R.N., Anokhin A.P. (2012). Human gut microbiome viewed across age and geography. Nature.

[64] Benezra A., DeStefano J., Gordon J.I. (2012). Anthropology of microbes. Proc. Natl. Acad. Sci. USA.

[65] Li J., Quinque D., Horz H.-P., Li M., Rzhetskaya M., Raff J.A., Hayes M.G., Stoneking M. (2014). Comparative analysis of the human saliva microbiome from different climate zones: Alaska, Germany, and Africa. BMC Microbiol..

[66] Henne K., Li J., Stoneking M., Kessler O., Schilling H., Sonanini A., Conrads G., Horz H.-P. (2014). Global analysis of saliva as a source of bacterial genes for insights into human population structure and migration studies. BMC Evol. Biol..

[67] Nasidze I., Li J., Schroeder R., Creasey J.L., Li M., Stoneking M. (2011). High diversity of the saliva microbiome in Batwa Pygmies. PLoS ONE.

[68] Nam Y.-D., Chang H.-W., Kim K.-H., Roh S.W., Kim M.-S., Jung M.-J., Lee S.-W., Kim J.-Y., Yoon J.-H., Bae J.-W. (2008). Bacterial, archaeal, and eukaryal diversity in the intestines of Korean people. J. Microbiol. Seoul Korea.

[69] Tyakht A.V., Kostryukova E.S., Popenko A.S., Belenikin M.S., Pavlenko A.V., Larin A.K., Karpova I.Y., Selezneva O.V., Semashko T.A., Ospanova E.A. (2013). Human gut microbiota community structures in urban and rural populations in Russia. Nat. Commun..

[70] Pitt P., de Bruijn K.M., Beeching M.F., Goldberg E., Blendis L.M. (1980). Studies on breath methane: The effect of ethnic origins and lactulose. Gut.

[71] Segal I., Walker A.R., Lord S., Cummings J.H. (1988). Breath methane and large bowel cancer risk in contrasting African populations. Gut.

[72] Le Marchand L., Wilkens L.R., Harwood P., Cooney R.V. (1993). Breath hydrogen and methane in populations at different risk for colon cancer. Int. J. Cancer J. Int. Cancer.

[73] Nava G.M., Carbonero F., Ou J., Benefiel A.C., O’Keefe S.J., Gaskins H.R. (2012). Hydrogenotrophic microbiota distinguish native Africans from African and European Americans: Diet and colonic hydrogenotrophs. Environ. Microbiol. Rep..

[74] Carbonero F., Benefiel A.C., Gaskins H.R. (2012). Contributions of the microbial hydrogen economy to colonic homeostasis. Nat. Rev. Gastroenterol. Hepatol..

